# Human T-lymphotropic virus 1 and 2 among people who used illicit drugs in the state of Pará, northern Brazil

**DOI:** 10.1038/s41598-019-51383-7

**Published:** 2019-10-14

**Authors:** Aldemir B. Oliveira-Filho, Ana Paula S. Araújo, Andreia Polliana C. Souza, Camila M. Gomes, Gláucia C. Silva-Oliveira, Luísa C. Martins, Benedikt Fischer, Luiz Fernando A. Machado, Antonio Carlos R. Vallinoto, Ricardo Ishak, José Alexandre R. Lemos, Emil Kupek

**Affiliations:** 1 Instituto de Estudos Costeiros, Universidade Federal do Pará, Bragança, PA Brazil; 2 Residência Multiprofissional em Saúde da Mulher e da Criança, Hospital Santo Antonio Maria Zaccaria, Universidade Federal do Pará, Bragança, PA Brazil; 3 Núcleo de Medicina Tropical, Universidade Federal do Pará, Belém, PA Brazil; 4 Faculty of Medical and Health Sciences, University of Auckland, Grafton, AK New Zealand; 5 Department of Psychiatry, University of Toronto, Toronto, ON Canada; 6 Departamento de Psiquiatria, Universidade Federal de São Paulo, São Paulo, SP Brazil; 7 Instituto de Ciências Biológicas, Universidade Federal do Pará, Belém, PA Brazil; 8 Centro de Hemoterapia e Hematologia do Pará, Belém, PA Brazil; 9 Departamento de Saúde Pública, Universidade Federal de Santa Catarina, Florianópolis, SC Brazil; 10 Programa de Pós-Graduação em Saúde Coletiva, Universidade Federal de Santa Catarina, Florianópolis, SC Brazil

**Keywords:** HTLV, Risk factors, Viral infection, Epidemiology

## Abstract

People who used illicit drugs (PWUDs) represent an important population group for acquisition and viral dispersion. In Brazil, high rates of the human T lymphotropic virus 1 (HTLV-1) and 2 (HTLV-2) have been reported in epidemiological studies. However, the epidemiological scenario of HTLV-1/2 infections in PWUDs is still poorly understood. Thus, this cross-sectional study determined the prevalence, frequency of subtypes and factors associated with HTLV-1/2 infections among PWUDs in the Brazilian state of Pará, an area considered endemic for this virus and with poor health services. Among 826 PWUDs, 53 (6.4%) presented anti-HTLV-1/2 antibodies by EIA and 44 (5.3%) presented proviral DNA by PCR. HTLV-1 and HTLV-2 were detected in 25 (3.0%) and 19 (2.3%) PWUDs, respectively. Subtypes 1a (25/44), transcontinental (23/44) and Japanese subgroups (2/44), 2b (6/44) and 2c (13/44) were identified. Involvement in illicit/criminal activity, daily use of illicit drugs, illicit drug use over 12 years, unprotected sex with other PWUDs, changes in genitalia (including ulcers and wounds), and more than 12 sexual partners were associated with HTLV-1/2 in PWUDs. This high prevalence and intense circulation of subtypes and subgroups of HTLV-1/2 is very worrying, and indicate the need for urgent actions for its control, prevention and treatment.

## Introduction

The human T-lymphotropic virus (HTLV) belongs to the family Retroviridae, subfamily Oncovirinae, and genus Deltaretrovirus. There are four types (HTLV-1, HTLV-2, HTLV-3 and HTLV-4), but only HTLV-1 and HTLV-2 are associated with chronic infections in humans, and more than 95% of infected individuals remain as asymptomatic carriers for the duration of their lives^1,2^. Based on the nucleotide diversity of its long terminal repeat (LTR) region, HTLV-1 has been classified in seven genetic subtypes (1a-1g), and HTLV-2 in four subtypes (2a-2d)^1,3,4^. HTLV-1 and HTLV-2 infections are widely distributed worldwide, with high or moderate endemicity in some regions such as southwestern Japan, sub-Saharan Africa, and specific areas of Iran and Melanesia^2–4^. In the Americas, high rates were recorded in Brazil and in Caribbean countries^1–3^. It is estimated that there are 2.5 million Brazilians infected with HTLV, and the highest rates were detected in the Brazilian states of Bahia, Maranhão and Pará^5^. In Brazil, HTLV-1a or Cosmopolitan subtype Transcontinental subgroup, HTLV-2a and HTLV-2c have shown high frequencies in different populations^1^.

HTLV-1/2 can be transmitted through the transfusion of contaminated blood or blood products, unprotected sexual contact, sharing of contaminated syringes and other instruments, or via transmission from mother to child^6,7^. People who use illicit drugs (PWUDs) represent an important population group for acquisition and viral dispersion, such as HTLV-1 and HTLV-2^8^. Infections with HTLV-1/2 have been recorded in PWUDs in different countries, mainly in people who have used injection drugs^9–11^. In Brazil, the number of PWUDs has increased significantly in recent decades. Crack cocaine is the main illicit drug consumed by Brazilian users, and has become a major public health problem^12^. To date, there is still limited information on HTLV infections in PWUDs in Brazil. In the state of Rio Grande do Sul (Southern Brazil), injecting cocaine users were five times more likely to be infected with HTLV-1/−2 than non-injecting users of cocaine^11^.

The Brazilian epidemiological scenario presents numerous social, economic and geographical differences that may facilitate the spread of viruses, such as HTLV-1/2. Among the regions, northern Brazil is notable for containing a significant portion of the Amazon rainforest. Historically, this area is an important route for trafficking in illicit drugs due to the geographical characteristics that make it difficult to control and facilitate the transportation and commercialization of drugs produced in South American countries^12–14^. The intense flow of people and illicit products associated with the lack of infrastructure and equipment for collective use has produced several problems in northern Brazil, such as abuse and sexual exploitation of children and adolescents, prostitution, consumption and trafficking of illicit drugs^13,15,16^. Recently, studies have reported a high prevalence of adolescents and young adults who used illicit drugs^15,17^. In northern Brazil, asymptomatic infections and diseases associated with HTLV-1/2 have already been reported in different groups: blood donors, pregnant women, Japanese immigrants, riverside people, and indigenous from more than 25 Amazonian tribes^18–25^. However, the epidemiological scenario of HTLV infections in PWUDs is still unknown in this immense Brazilian region, as well as in other vulnerable groups. Thus, this study determined the prevalence of HTLV-1/2 infections, the frequency of viral subtypes, and the factors associated with these infections among PWDUs in the Brazilian state of Pará, an area considered endemic for HTLV-1/2 and with poor health services.

## Results

### Snowball chain length

**Figure 1:**
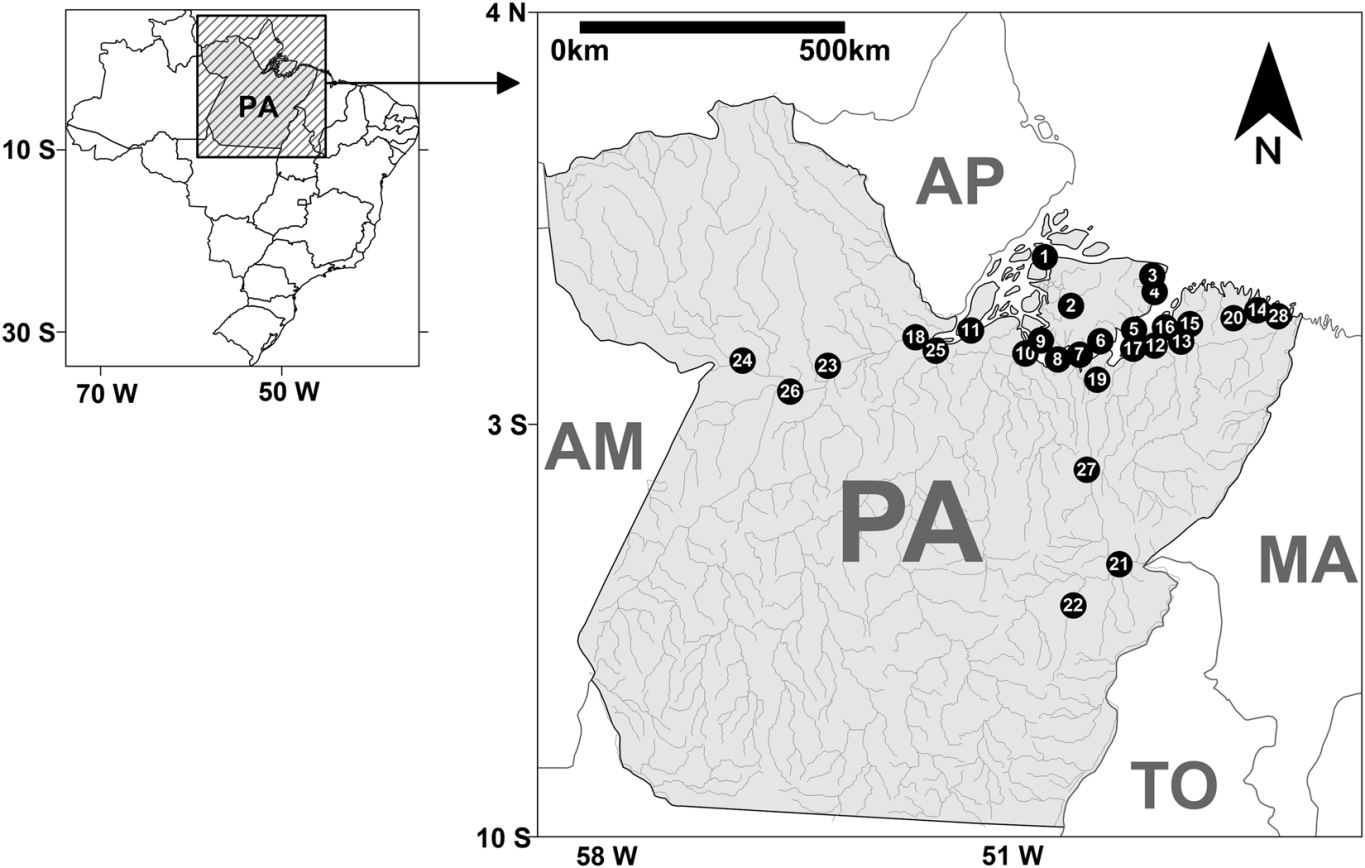
Geographic location of collection points and information of people who used illicit drugs in 28 municipalities in the state of Pará (PA), northern Brazil. Points = municipalities: (1) Afuá*; (2) Anajás*; (3) Soure*; (4) Salvaterra*; (5) Ponta de Pedras*; (6) São Sebastião da Boa Vista*; (7) Curralinho*; (8) Bagre*; (9) Breves; (10) Melgaço*; (11) Gurupá*; (12) Belém; (13) Benevides; (14) Bragança; (15) Castanhal; (16) Marituba; (17) Abaetetuba; (18) Almeirim*; (19) Cametá; (20) Capanema; (21) Marabá; (22) Parauapebas; (23) Altamira; (24) Óbidos; (25) Porto de Moz*; (26) Santarém; (27) Tucuruí; (28) Augusto Correa*. *Municipality considered small (<50,000 inhabitants).

In this study, 907 PWUDs were accessed, but 81 PWUDs were excluded (49 had <18 years old and 32 used illicit drugs <3 months). In total, the sample number of this study was 826 PWUDs. Samples and information from PWUDs were collected in all regions in the Brazilian state of Pará (Fig. 1). The average PWUDs in each municipality was 29 (standard deviation = ±20). The highest and lowest number of PWUDs was obtained in Belém (n = 102) and Gurupá (n = 12), respectively (Table S1).

### Characteristics of PWUDs and subgroups

In this study sample (n = 826 PWUDs), most were men, young, singles, had up to 10 years of education, had a reduced monthly income, and declared themselves to be heterosexual (Table 1). All PWUDs declared the frequent use of non-injecting drugs in the last 12 months. However, 675 (81.7%) reported having used more than one illicit drug in their lifetime and were classified as poly-users. Crack, called oxy, was the main drug used (48.4%). However, 111 (13.4%) PWUDs reported having used injectable cocaine at least once in their lifetime, and most of them had used illicit drugs more than 12 years. Based on the route of administration, PWUDs were divided into two subgroups: people who used only non-injecting drugs (NIDUs) and those used both injecting drugs and non-injecting (IDUs). These subgroups presented significant variation regarding their characteristics/behavior (Table 1).

**Table 1:** Key characteristics or behaviors of people who used illicit drugs and their subgroups.

Characteristics/behaviors	715 NIDUs	111 UDIs	826 PWUDs	p-value*
	n (%)	n (%)	n (%)	
**Gender**				
Male	519 (72.6)	85 (76.6)	604 (73.1)	0.53
Female	192 (26.9)	26 (23.4)	218 (26.4)	
Transgendered	4 (0.5)	—	4 (0.5)	
**Age**				
18–29 years	443 (62.0)	26 (23.4)	469 (56.8)	<0.01
30–39 years	202 (28.2)	45 (40.6)	247 (29.9)	
40+ years	70 (9.8)	40 (36.0)	110 (13.3)	
**Sexual orientation**				
Heterosexual	650 (90.9)	103 (92.8)	753 (91.2)	0.51
Same-sex (including bisexual)	65 (9.1)	8 (7.2)	73 (8.8)	
**Marital status** ^**†**^				
Single, separated or widowed	472 (66.0)	78 (70.3)	550 (66.6)	0.38
Married or co-habitating	243 (34.0)	33 (29.7)	276 (33.4)	
**Education**				
Some years in elementary school (including illiterates)	392 (54.8)	67 (60.4)	459 (55.6)	0.27
Completed elementary school or higher	323 (45.2)	44 (39.6)	367 (44.4)	
**Monthly income (Brazilian minimum wage)** ^**†**^				
≤1 minimum wage	465 (65.0)	40 (36.0)	515 (62.3)	<0.01
2 to 3 times minimum wage	234 (32.7)	37 (33.3)	271 (32.8)	
>3 times minimum wage	16 (2.3)	34 (30.7)	40 (4.9)	
**Main income source** ^**†**^				
Regular or irregular work	447 (62.5)	58 (52.3)	505 (61.1)	0.01
Social benefits or pension	95 (13.3)	12 (10.8)	107 (13.0)	
Criminal/illicit activity	173 (24.2)	41 (36.9)	214 (25.9)	
**Main illicit drug used** ^**†**^				
Marijuana	74 (10.3)	—	74 (9.0)	<0.01
Crack	357 (49.9)	43 (38.7)	400 (48.4)	
Cocaine (powder or paste)	284 (39.7)	68 (61.3)	352 (42.6)	
**Frequency of illicit drug use** ^**†**^				
Daily	487 (68.1)	80 (72.1)	567 (68.6)	<0.01
A few times a week	160 (22.4)	31 (27.9)	191 (23.1)	
A few times a month or rarely	68 (9.5)	—	68 (8.3)	
**Period of illicit drug use**				
≤5 years	197 (27.6)	2 (1.8)	199 (24.1)	<0.01
6 to 11 years	299 (41.8)	28 (25.2)	327 (39.6)	
12+ years	219 (30.6)	81 (73.0)	300 (36.3)	
Sharing of drug use paraphernalia^†^	407 (56.9)	79 (71.2)	486 (58.8)	<0.01
Unprotected sex (vaginal and/or anal)^†^	521 (72.9)	91 (82.0)	612 (74.1)	0.02
Unprotected sex with other PWUDs^†^	458 (64.1)	43 (38.7)	501 (60.7)	<0.01
Changes in genitalia (including ulcers and wounds)^†^	229 (32.0)	42 (37.8)	271 (32.8)	0.23
Sex exchange for money or drugs^†^	359 (50.2)	44 (39.6)	403 (48.8)	0.04
**Number of sexual partners** ^**†**^				
Up to 5 partners	105 (14.7)	19 (17.1)	124 (15.0)	0.05
6 to 12 partners	263 (36.8)	52 (46.9)	315 (38.1)	
>12 partners	347 (48.5)	40 (36.0)	387 (46.9)	
Blood transfusion	91 (12.7)	10 (9.0)	101 (12.2)	0.27
Tattoos	477 (66.7)	78 (70.3)	555 (67.2)	0.46

PWUDs: People who used illicit drugs. NIDUS: people used only non-injecting drugs during their lives. IDUs: People who have used injectable and non-injectable drugs during their lives. Average of Brazilian minimum wage equals 800 Reais (equivalent to 210 US dollars) from 2013 to 2018. ^†^Last 12 months. *Calculated by the Chi-square test.

### Diagnosis of HTLV-1/2 infections

Overall, 53 (6.4%) PWUDs presented anti-HTLV-1/2 antibodies using EIA and 44 (5.3%) PWUDs presented proviral DNA using different PCR protocols. There was no disagreement between the results provided by the two HTLV proviral DNA detection protocols. All samples with anti-HTLV-1/2 antibodies by EIA, submitted to DNA isolation, presented positive results for the presence of fragment of the albumin gene, indicating the success in obtaining genetic material. Proviral DNA was not detected in nine seropositive samples, possibly indicative of non-specific seropositivity. Among PWUDs with proviral DNA, most of them (59.1%) were living in small municipalities in the state of Pará (<50,000 inhabitants). HTLV-1 and HTLV-2 were detected in 25 (3.0%) and 19 (2.3%) PWUDs, respectively (Table 2). All PWUDs infected with HTLV-1/2 were asymptomatic carriers; consequently, none of them were under medical supervision. All HTLV-infected PWUDs were unaware of their condition prior to this study.

**Table 2:** Frequency of exposure markers, subtypes and subgroups of HTLV-1/2 in people who used illicit drugs and their subgroups in the state of Pará, northern Brazil.

Exposure markers	% (Positive/Total)		
	NIDUs	IDUs	PWUDs
Anti-HTLV-1/2 antibodies	3.9 (28/715)	22.5 (25/111)	6.4 (53/826)
HTLV-1/2 proviral DNA	2.8 (20/715)	21.6 (24/111)	5.3 (44/826)
HTLV-1 proviral DNA	2.1 (15/715)	9.0 (10/111)	3.0 (25/826)
HTLV-2 proviral DNA	0.7 (5/715)	12.6 (14/111)	2.3 (19/826)
**Subtypes and subgroups**			
1a (Cosmopolitan)	75.0 (15/20)	41.7 (10/24)	56.8 (25/44)
A (Transcontinental)	75.0 (15/20)	33.4 (8/24)	52.3 (23/44)
B (Japanese)	—	8.3 (2/24)	4.5 (2/44)
2b	10.0 (2/20)	16.6 (4/24)	13.7 (6/44)
2c	15.0 (3/20)	41.7 (10/24)	29.5 (13/44)

PWUDs: People who used illicit drugs. NIDUs: people used only non-injecting drugs during their lives. IDUs: People who have used injectable and non-injectable drugs during their lives.

### HTLV-1/2 subtypes and subgroups

**Figure 2:**
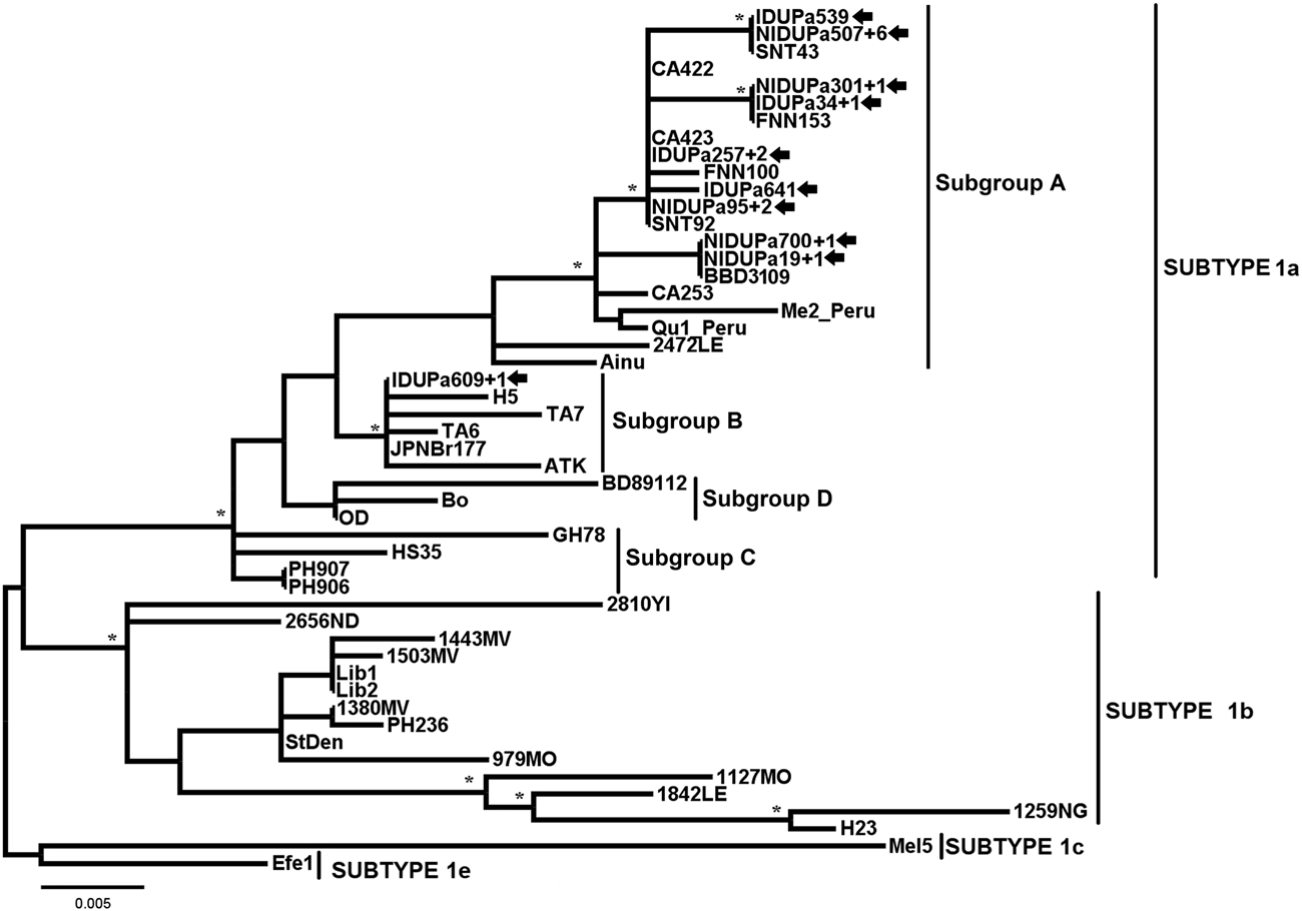
Rooted phylogenetic tree, showing the evolutionary relationship of human T-lymphotropic virus 1 strains, including the strains detected among people who used illicit drugs in the Brazilian state of Pará (indicated by arrows). The tree was constructed by the Bayesian method after alignment of 409 nucleotides of the 5′ LTR. The statistical support was applied using 1000 bootstrap replicates. *Boostrap values: ≥90% and posterior probabilities ≥0.95.

HTLV-1 predominated in PWUDs and also in the subgroup NIDUs (Table 2). The 25 samples of HTLV-1 were clustered with strains of subtype 1a (Cosmopolitan) (Table 2 and Fig. 2), of which 23 samples (15 NIDUs and 8 IDUs) were classified into the subgroup A (Transcontinental) and 2 (2 IDUs) into the subgroup B (Japanese). The Japanese subgroup samples (IDUPa609 + 1) belonged to young PWUDs of Japanese descended from the municipality of Tomé-Açu in the state of Pará. During the construction of the HTLV-1 phylogenetic tree, 10 of 25 sequences showed some nucleotide difference, so only one copy of each identical nucleotide sequence remained in alignment. In Fig. 2, eight distinct clusters were detected, each showing the total number of sequences (e.g., NIDUPa507 + 6 = sequence from the NIDU Pa537 and other 6 identical sequences from PWUDs).

**Figure 3:**
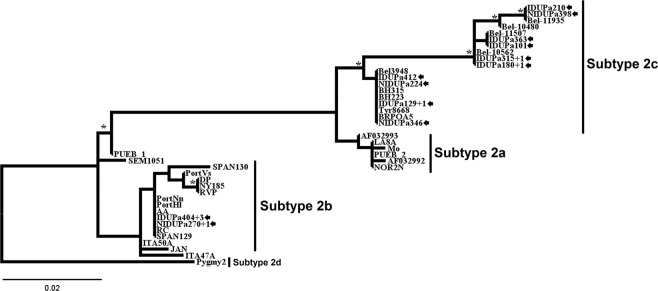
Rooted phylogenetic tree, showing the evolutionary relationship of human T-lymphotropic virus 2 strains, including the strains detected among people who used illicit drugs in the Brazilian state of Pará (indicated by arrows). The tree was constructed by the Bayesian method after alignment of 408 nucleotides of the 5’ LTR. The statistical support was applied using 1000 bootstrap replicates. *Boostrap values: ≥90% and posterior probabilities ≥0.95.

On the other hand, the HTLV-2 predominated among IDUs. The HTLV-2 samples were grouped into two strain subtypes: 2b (13.7%) and 2c (29.5%) (Table 2 and Fig. 3). HTLV-2b strains were identified in 2 NIDUs and 4 IDUs (Table 2 and Fig. 3). HTLV-2c strains were identified in 10 IDUs and 3 NIDUs (Table 2 and Fig. 3). Among the PWDUs identified with subtypes 2b, one of PWUDs (IDUPa404 + 3) reported having resided in Peru for almost two years, the period during which he injected drugs, shared the drug use equipment, used non-injected drugs, and had unprotected sex. Among the PWDUs identified with subtypes 2c, two (IDUPa180 + 1 and IDUPa315 + 1) of them worked in river navigation companies in the Amazon (river routes traveled through the municipalities of Belém, Breves, Óbidos, Monte Alegre, Santarém, Manaus Curralinho and Santana) for more than 5 years. During the construction of the HTLV-2 phylogenetic tree, 12 of 19 sequences showed some nucleotide difference. Thus, only one copy of each identical nucleotide sequence remained in alignment. In Fig. 3, five distinct clusters were detected, each showing the total number of sequences (e.g., IDUPa404 + 3 = sequence from the IDU Pa404 and other 3 identical sequences from PWUDs). No significant differences were detected between the NIDUs and IDUs subgroups for the HTLV-1 strains (Transcontinental and Japanese) and the HTLV-2 subtypes (2b and 2c). The information used for the construction of phylogenetic trees is listed in Table S2. Finally, further information on the distinct clusters in Figs 2 and 3 are listed in Table S3.

### Factors associated with HTLV-1/2

Bivariate analysis indicated six risk behaviors associated with HTLV-1/2 in PWUDs: involvement in illicit/criminal activity in the last 12 months, daily use of illicit drugs in the last 12 months, illicit drug use over 12 years, unprotected sex with other PWUDs in the last 12 months, changes in genitalia (including ulcers and wounds) in the last 12 months, and more than 12 sexual partners in the last 12 months (Table 3). These same risk behaviors were also associated with HTLV-1/2 by multivariate analysis (Table 4). Statistical analysis indicated that three of these six factors are also associated with HTLV-1/2 in the NIDUs and IDU subgroups. In NIDUs, the main use of crack in the last 12 months, daily use of illicit drugs in the last 12 months, unprotected sex (vaginal and/or anal) in the last 12 months, changes in genitalia (including ulcers and wounds) in the last 12 months, and sex exchange for money or drugs in the last 12 months were also associated with HTLV-1/2 in both bivariate and multivariate analysis (Tables 3 and 4). In IDUs, the main use of cocaine (powder or paste) in the last 12 months, sharing of drug use paraphernalia in the last 12 months, and drug use over 12 years or longer were also associated with HTLV-1/2 using bivariate and multivariate analysis (Tables 3 and 4). The factors not associated (p > 0.05) with HTLV-1/2 are listed in Table S4.

**Table 3:** Characteristics/behaviors associated with HTLV-1/2 in people who use illicit drugs and their subgroups using bivariate analysis.

Characteristics/behaviors	715 NIDUs			111 IDUs			826 PWUDs		
	*n* NIDUs	*n* PCR+	OR (95% CI)	*n* IDUs	*n* PCR+	OR (95% CI)	*n* PWUDs	*n* PCR+	OR (95% CI)
Criminal/illicit activity^†^	173	10	3.3 (1.3–8.0)*	41	15	3.9 (1.5–10.1)*	214	25	4.1 (2.2–7.7)*
Main use of crack^†^	357	18	9.5 (2.2–38.1)*	43	5	0.3 (0.1–1.0)	400	23	1.2 (0.6–2.2)
Main use of cocaine (powder or paste)^†^	284	7	0.8 (0.3–2.1)	68	20	4.0 (1.3–12.9)*	352	27	1.3 (0.7–2.4)
Sharing of drug use paraphernalia^†^	407	9	0.6 (0.3–1.5)	79	22	5.8 (1.3–23.4)*	486	31	1.7 (0.9–3.3)
Daily use of illicit drugs^†^	487	19	9.2 (1.2–57.3)*	80	19	1.6 (0.5–4.7)	567	38	3.0 (1.3–7.3)*
Drug use over 12 years	219	3	0.4 (0.1–1.4)	81	23	11.5 (1.5–69.4)*	300	26	2.7 (1.4–5.0)*
Unprotected sex (vaginal and/or anal)^†^	511	19	7.8 (1.1–56.2)*	91	18	0.6 (0.2–1.7)	602	37	2.0 (0.9–4.6)
Unprotected sex with other PWUDs^†^	458	19	11.1 (1.5–61.2)*	43	15	3.5 (1.4–9.0)*	501	34	2.3 (1.1–4.7)*
Changes in genitalia^†^	229	15	8.3 (3.0–21.2)*	42	12	1.9 (0.7–4.6)	271	27	3.3 (1.7–6.0)*
Sex exchange for money or drugs^†^	359	18	9.3 (2.2–38.6)*	44	7	0.6 (0.2–1.5)	403	25	1.4 (0.8–2.6)
More than 12 sexual partners^†^	347	16	4.4 (1.5–13.1)*	40	15	4.1 (1.6–10.6)*	387	31	2.9 (1.5–5.6)*

PWUDs: People who used illicit drugs. NIDUs: people used only non-injecting drugs during their lives. IDUs: People who have used injectable and non-injectable drugs during their lives. ^†^Last 12 months. OR: Odds ratio. CI: Confidence Intervals. *p-value < 0.05.

**Table 4:** Characteristics/behaviors associated with HTLV-1/2 in people who use illicit drugs and their subgroups using multivariate analysis.

Characteristics/Behaviors	Multiple Logistic Regression		Hosmer-Lemeshow Test
Identified in NIDUs	*p-value*	aOR (95% CI)	_HL_χ^2^ (*p-value*)
Criminal/illicit activity^†^	0.01	2.9 (1.2–6.3)	7.1 (0.4)
Main use of crack^†^	<0.01	10.1 (3.3–34.5)	
Daily use of illicit drugs^†^	<0.01	9.5 (1.5–48.4)	
Unprotected sex (vaginal and/or anal)^†^	<0.01	8.0 (1.4–43.8)	
Unprotected sex with other PWUDs^†^	<0.01	11.6 (1.7–52.6)	
Changes in genitalia^†^	<0.01	8.8 (3.1–23.7)	
Sex exchange for money or drugs^†^	<0.01	8.2 (2.3–31.7)	
More than 12 sexual partners^†^	<0.01	5.8 (1.4–17.3)	
**Identified in IDUs**	***p-value***	**aOR (95% CI)**	_**HL**_ **χ** ^**2**^ **(** ***p-value*** **)**
Criminal/illicit activity^†^	<0.01	4.6 (1.8–12.3)	4.2 (0.5)
Main use of cocaine (powder or paste)^†^	<0.01	4.2 (1.5–14.3)	
Sharing of drug use paraphernalia^†^	<0.01	6.2 (1.9–25.1)	
Drug use over 12 years	<0.01	8.3 (1.6–29.1)	
Unprotected sex with other PWUDs^†^	0.03	2.9 (1.3–8.2)	
More than 12 sexual partners^†^	0.02	3.7 (1.3–9.7)	
**Identified in PWUDs**	***p-value***	**aOR (95% CI)**	_**HL**_ **χ** ^**2**^ **(** ***p-value*** **)**
Criminal/illicit activity^†^	<0.01	3.2 (1.7–7.0)	4.1 (0.5)
Daily use of illicit drugs^†^	0.01	2.8 (1.2–6.1)	
Drug use over 12 years	0.02	2.4 (1.3–5.1)	
Unprotected sex with other PWUDs^†^	<0.01	3.0 (1.4–4.9)	
Changes in genitalia^†^	<0.01	3.1 (1.5–5.7)	
More than 12 sexual partners^†^	<0.01	3.9 (1.4–6.1)	

PWUDs: People who used illicit drugs. NIDUs: people used only non-injecting drugs during their lives. IDUs: People who have used injectable and non-injectable drugs during their lives. ^†^Last 12 months. aOR: adjusted Odds ratio. CI: Confidence Intervals.

## Discussion

This study is the first epidemiological report on the HTLV-1/2 in PWUDs from the Amazon region. In a large sample of hard-to-reach people from a variety of sites and good geographical coverage in a remote area of Brazil, several key characteristics were identified and associated with acquisition and spread of HTLV-1/2. In general, the characteristics of the PWUDs are consistent with the findings of other studies conducted in Brazil^11–14,26^. Moreover, the differences between NIDUs and IDUs were also identified. Initially, the route of administration of illicit drugs was the main difference but other distinct features relevant to the HTLV spread were also established.

The seroprevalence of HTLV-1/2 found in PWUDs and its subgroups NIDUs and IDUs are higher than those reported in other studies conducted in HTLV endemic areas in Brazil (states of Bahia, Maranhão and Pará – 0.3% to 1.8%)^22,27–30^. The seroprevalences detected in this study are relatively similar to those found in studies conducted with IDUs in different countries such as Argentina (19.1%), the Dominican Republic (6.0%), Sweden (3.2%) and Spain (3.5% to 6.2%)^31–34^. This is indicative of the influence of the drug use profile on the potential exposure to HTLV-1/2 of the present study.

Different epidemiological characteristics of HTLV-1/2 have been reported in geographic areas and specific population groups^2–4^. HTLV-1 has a worldwide distribution, but it is considered endemic in Brazil with low prevalence in the general population^3,5,35^. A geographic gradient has been reported in blood donors with lower prevalence in the south and rising to the north^21,35^. In the state of Bahia, an endemic area in northeastern Brazil, high rates were observed in women and in people over 50 years^27^, as well as in men who have sex with men and illicit drug users^11,36^. In this study, the predominance of HTLV-1 observed in PWUDs and NIDUs can be considered an example of the endemicity of this viral type reported in numerous Brazilian studies, and especially in the state of Pará – an endemic area in northern Brazil^22,28^. On the other hand, the HTLV-2 is endemic among IDUs in the United States, Europe, and Asia^10,33,34,37–39^, and particularly among indigenous tribes of different linguistic families inhabiting the Brazilian Amazon, where subtype 2c was described for the first time^1,19,23,24^. The predominance of HTLV-2 among IDUs in this study corroborates this information.

The findings of this study also indicate an intense circulation of the HTLV-1/2 subtypes and subgroups among PWUDs. The subtype 1a (Cosmopolitan) was predominant among PWUDs being also detected the predominance of subgroup A (Transcontinental). This HTLV-1 subtype and this HTLV-1 subgroup of are very common in northern Brazil^20–22,28^. Two PWUDs with the Japanese subgroup were descendants of Japanese immigrants from the municipality of Tomé-Açú, one of the colonies of Japanese migrants from the region of Kyushu in southeastern Japan. The presence of this HTLV-1 subgroup was also registered in another epidemiological investigation with 168 Japanese immigrants living in the municipality of Tomé-Açú^20^. Another interesting fact observed in this study was the high frequency of the HTLV-2 subtypes. In South America, the HTLV-2 predominates among indigenous groups, with subtype 2b clearly prevailing in Amerindian populations, except in Brazil where the subtype 2c predominates^1,25^. The latter is endemically distributed in the Amazon region and maintained under continuous transmission (vertical and horizontal) between members of different indigenous communities, being also detected on a smaller scale in urban populations^21,23,24^. In endemic populations, the HTLV-2b replication pattern exhibits high proviral loads resulting from extensive proliferation of infected cells^40^. The predominance of this subtype in NIDUs and IDUs has already been reported in Brazil^1^. Brazil has received both intense internal and external migration from Africa, Europe and Asia over the past five centuries. As a consequence, different HTLV subtypes and subgroups were introduced and distributed in the Brazilian population over the years^1,23,24^.

The findings of this study clearly demonstrate the contribution of the migratory process to the dispersion of HTLV-1/2 among the PWUDs in the state of Pará and possibly among the general population in the Amazon region. The latter hypotheses is strongly supported by the association between HTLV-1/2 clusters in phylogenetic trees with the profile of illicit drug use and risky sexual behavior. Accurate phylodynamic analysis using other genomic regions and more epidemiological details of PWUDs will be done in the future in order to evaluate the epidemic potential and effective targeting of preventive actions.

Overall, this study shows the HTLV-1/2 acquisition and spread among PWUDs occur mainly through sexual contact. This is most evident in NIDUs due to the HTLV-1 predominance among them. Daily and long-term use of crack is associated prostitution and unprotected sex with multiple partners (within and outside of the PWUD groups), all of which facilitate the acquisition and spread of HTLV-1. The same goes for the HTLV-2, although on a smaller scale. This scenario corroborates the assertion that many factors are associated with HTLV shedding and HTLV proviral load in genital (seminal and vaginal) secretions^7^. HTLV-infected lymphocytes are considered the primary vectors of sexual transmission^41^. The presence of inflammation or sores on the genitals and sexually transmitted infection may facilitate the HTLV transmission because these conditions expose target cells through genitomucous lesions^7,41^. Unprotected sex associated with lesions on the genitalia of PWUDs was an important contributor to HTLV-1/2 spread and possibly other pathogens (e.g. *Treponema pallidum*), similar to that reported in the Brazilian state of Bahia - high number of HTLV-1 infections associated with *T. pallidum* infection through the sexual route^27^.

The pattern of illicit drug use enhances the risk of exposure to the HTLV-1/2. This is most evident in IDUs due to the predominance of HTLV-2. In IDUs, however, the HTLV-1/2 spread occurs through both sexual and parenteral route. Long-term cocaine use (including injectable use) associated with unprotected sex and multiple sexual partners have enabled the spread of HTLV-1/2, especially the HTLV-2. In Brazil, injecting drug use, sharing of drug use paraphernalia and unprotected sex with IDUs have already been reported as factors associated with HTLV-1/2 infection. The use of injecting drugs has also been indicated as an important factor associated with the transmission of HTLV-2 in Brazil, Argentina and Spain^11,31,34,42^. Involvement with illicit/criminal activities facilitates greater contact with drug traffickers in the Amazon region and therefore enables access to a greater quantity and diversity of drugs, thus contributing significantly to chemical dependence and multifactorial exposure to the HTLV-1/2 and other pathogens.

The present study points to several key implications for public health intervention to prevent the HTLV transmission in the Amazon region. First, none of the PWDUs were aware of HTLV-1/2 infection status and therefore were not under medical supervision. This points to a major deficiency in the state of Pará regarding the availability of laboratory HTLV-1/2 tests. This situation needs to be urgently addressed through improved required resources, services and technical skills because the diagnosis is an essential tool for surveillance, prevention and clinical monitoring, especially in an endemic area. Furthermore, it points to the need for improved targeted prevention concerning HTLV-related risks. Given that primary risk factors for HTLV identified risky sexual behavior, there is evidently an urgent need for improved prevention of unprotected sex through targeted education and distribution of prevention resources or materials (e.g. condoms). It will furthermore be important to understand to which extent the common sexual risk behaviors in the study population are directly associated with behavioral or economic motivation of drug use or procurement, as this will determine the need for other or more specific prevention targets/measures (e.g. drug dependence treatment) towards reducing HTLV risk behavior. Overall, considering the duration and intensity of illicit drug use reported by PWUDs in this study, most participants probably require treatment for chemical dependence/disorders^43^. Mental comorbidities are disproportionately common in people who regularly used illicit drugs^43,44^. A program for drug treatment, including the availability of resources to minimize the harm associated with the use of illicit drugs, should be implemented. The availability and access to diagnosis, prevention and treatment must be improved urgently in the state of Pará.

This study has limitations and should be considered. The study restriction to 28 of the 147 municipalities of the state of Pará was the first limiting factor, indicative that the sample may not represent the population of PWUDs. Second, PWUDs who initiated illicit drug use during adolescence were excluded by the age limit of 18 years or older. In addition, there are other alternatives for sampling hidden populations as respondent-driven sampling^45^. Confidential information on criminal or illicit activities such as trafficking and drug use or risky sexual behavior could have omitted by PWUDs while providing information. Furthermore, HTLV-1/2 screening used EIA, so recent infections with small concentration of anti-HTLV-1/2 antibodies may have not been detected, thus resulting in false negative test result, as previously reported in the Amazon region^46^. Finally, the ability to establish causality is limited in cross-sectional study.

This study is unique in bringing together phylogenetic, epidemiological and behavioral risk factors for the HTLV-1/2 transmission in the Amazon region. High HTLV-1/2 prevalence was found among PWUDs in the Brazilian state of Pará, with a distinct predominance in NIDUs and IDUs. Sexual risk behaviors contributed significantly to the virus acquisition and spread within this vulnerable population, and also to the general population. The inefficiency of the health care system makes the epidemiological scenario even more worrying since there is an urgent need to implement actions for the control, prevention and treatment of the HTLV-infected people.

## Methods

### Study design and data collection

This cross-sectional study was based on biological and self-reported socio-behavioral data from a convenience sample of 826 PWUDs from 28 municipalities located in the state of Pará, northern Brazil (Fig. 1). All samples and participants’ personal information were accessed in a scientific study on viral infections in PWUDs in the Brazilian states of Amapá and Pará^47^.

### Laboratory tests

All PWUDs samples were tested for the presence of anti-HTLV-1/2 antibodies (Murex HTLV-I + II GE80/81, DiaSorin, UK) by enzyme-linked immunosorbent assay (EIA). EIA-positive samples were subjected to nucleic acid isolation using the Wizard Genomic DNA Purification Kit (Promega, USA). The presence of DNA was evaluated by *Albumin* gene fragment amplification (internal control) using real-time polymerase chain reaction (PCR)^48^. The presence of HTLV-1/2 DNA was evaluated using two protocols: (1) Amplification and detection of non-homologous fragments of the *pol* gene using real-time PCR^48^; and (2) Amplification of *pX* region fragments using Nested-PCR followed by enzymatic digestion with *Taq*I^49^. HTLV-1/2 infection was defined by the presence of proviral DNA by either of two methods.

### Sequencing and phylogenetic analysis

All samples with HTLV-1/2 DNA were subjected to Nested-PCR for fragment amplification of the 5′ LTR^49^. These fragments were subjected to 1.5% agarose gel electrophoresis and subsequently purified using a commercial kit (QIAquick PCR Purification Kit, Qiagen, USA). The nucleotide sequencing was performed using the BigDyeTerminator 3.1 kit (Applied Biosystems, USA) by capillary electrophoresis in the system (ABI PRISM 3130, Applied Biosystems, USA). The nucleotide sequences obtained were edited and aligned with the program CLUSTAL W, implemented in BioEdit software^50^. Based on Akaike’s (AIC) and Bayesian (BIC) information criteria, the most suitable phylogenetic cluster model for the HTLV-1 and HTLV-2 data was indicated by JModelTest2 software^51^. BIC was used in Bayesian inference using MrBayes 3.2.1 software^52^. AIC was used in the Maximum Likelihood (ML) and Maximum Parsimony (MP) analyzes using PAUP* 4.0b10 software^53^. The Bayesian analysis consisted of two independent runs with chain length of 2,000,000 MCMC runs each and 10% of burn-in, sampled every 1000 interactions. The robustness of the groups was evaluated using 1,000 bootstrap replicates. Topologies of the phylogenetic trees were visualized using TreeView software^54^. Reference sequences of the HTLV-1/2 subtypes, available from GenBank, were added in the alignment (Supplementary Material - Table S1). The sequences obtained in this study were deposited in GenBank (MN078967-MN079010).

### Statistical analysis

SPSS 20.0 for Windows was used to perform all statistical procedures. Chi-square test was used to identify significant differences between subgroups of PWUDs. Odds ratios (OR) and 95% confidence intervals (CI) were used as measures of the strength of association between HTLV-1/2 infections (outcome) and independent variables by logistic regression models (bivariate and multivariate)^47^. A p-value (p) < 0.05 significance value was considered for all analyses.

### Ethics statement

The present study was approved by the Ethics Committee on Research Involving Human Subjects of the Federal University of Pará, Brazil (CAAE: 37536314.4.0000.5172). All procedures performed in this study were in accordance with the relevant guidelines and regulations. All PWUDs were included after providing informed and written consent, and participants with positive results for HTLV-1/2 infections received counseling and were directed to care in the public health network.

## Supplementary information

SUPPLEMENTARY MATERIALS

## Data Availability

The datasets generated during and/or analyzed during the current study are available from the corresponding author on reasonable request.
