# Supplementary material for: Human T-lymphotropic virus 1 and 2 among people who used illicit drugs in the state of Pará, northern Brazil
Source: Sci Rep. 2019 Oct 14;9:14750. doi: 10.1038/s41598-019-51383-7 (PMC6791863; doi:10.1038/s41598-019-51383-7)
Supplement: Supplementary file 1 — SUPPLEMENTARY MATERIALS [file 41598_2019_51383_MOESM1_ESM.doc]

**SUPPLEMENTARY MATERIALS**

Title: Human T-lymphotropic virus 1 and 2 among people who used illicit drugs in the state of Pará, northern Brazil.

Authors: Aldemir B. Oliveira-Filho; Ana Paula S. Araújo; Andreia Polliana C. Souza; Camila M. Gomes; Gláucia C. Silva-Oliveira; Luísa C. Martins; Benedikt Fischer; Luiz Fernando A. Machado; Antonio Carlos R. Vallinoto; Ricardo Ishak; José Alexandre R. Lemos; Emil Kupek.

Journal: Scientific Reports.

Table S1. Sample of people who used illicit drugs (PWUIDs) accessed in each municipality of the Brazilian state of Pará.

| Municipality | Number in figure 1 | Regions in the state of Pará | Number of PWUIDs accessed |
| --- | --- | --- | --- |
| Afuá | 1 | Marajó | 15 |
| Anajás | 2 | Marajó | 16 |
| Soure | 3 | Marajó | 20 |
| Salvaterra | 4 | Marajó | 22 |
| Ponta de Pedras | 5 | Marajó | 18 |
| São Sebastião da Boa Vista | 6 | Marajó | 14 |
| Curralinho | 7 | Marajó | 32 |
| Bagre | 8 | Marajó | 18 |
| Breves | 9 | Marajó | 57 |
| Melgaço | 10 | Marajó | 16 |
| Gurupá | 11 | Marajó | 12 |
| Belém | 12 | Metropolitana de Belém | 102 |
| Benevides | 13 | Metropolitana de Belém | 30 |
| Bragança | 14 | Nordeste | 40 |
| Castanhal | 15 | Nordeste | 25 |
| Marituba | 16 | Metropolitana de Belém | 50 |
| Abaetetuba | 17 | Nordeste | 26 |
| Almeirim | 18 | Baixo Amazonas | 20 |
| Cametá | 19 | Nordeste | 17 |
| Capanema | 20 | Nordeste | 26 |
| Marabá | 21 | Sudeste | 38 |
| Parauapebas | 22 | Sudeste | 26 |
| Alltamira | 23 | Sudoeste | 58 |
| Óbidos | 24 | Baixo Amazonas | 19 |
| Porto de Moz | 25 | Baixo Amazonas | 16 |
| Santarém | 26 | Baixo Amazonas | 43 |
| Tucuruí | 27 | Sudeste | 28 |
| Augusto Correa | 28 | Nordeste | 20 |

Table S2. GenBank information and evolutionary models used for the construction of HTLV-1 and HTLV-2 phylogenetic trees.

| HTLV-1 | |  | HTLV-2 | |  |
| --- | --- | --- | --- | --- | --- |
| Strains | Access number | Evolutionary models | Strains | Access number | Evolutionary models |
| SNT43 | DQ070891 | TrN+G (AIC) and  HKY+G (BIC) | BRPOA5 | KX198746 | TPM2uf+G (AIC and BIC) |
| SNT92 | KY928524 |  | PortVs | AY622979 |  |
| FNN100 | DQ005547 |  | PortHl | L77242 |  |
| FNN153 | DQ005550 |  | Bel3948 | AF306724 |  |
| Qu1_Peru | Y16475 |  | Bel-11935 | AY920499 |  |
| Me2_Peru | Y16479 |  | Bel-11507 | AY920500 |  |
| TA6 | U53074 |  | Bel-10562 | GU573734 |  |
| TA7 | U53075 |  | Gty9272 | KY928552 |  |
| JPNBr177 | LC210070 |  | Tyr8668 | KY928551 |  |
| Lib1 | Y17016 |  | Mo | JN247459 |  |
| Lib2 | Y17017 |  | NOR2N | U10258 |  |
| Bo | U12804 |  | LA8A | JN247462 |  |
| OD | U12805 |  | PUEB_2 | JN247461 |  |
| GH78 | D23693 |  | Pygmy2 | Y14365 |  |
| PH236 | L76307 |  | SPAN130 | U10266 |  |
| StDen | L76306 |  | ITA47A | U10254 |  |
| PH906 | KJ544830 |  | ITA50A | AY442388 |  |
| PH907 | KJ544830 |  | NY185 | L77237 |  |
| BD89112 | DQ235698 |  | PUEB_1 | U10261 |  |
| Ainu | D23694 |  | SEM1051 | U10264 |  |
| Efe1 | Y17014 |  | SPAN129 | L77242 |  |
| H5 | M37299 |  | JAN | L77240 |  |
| H23 | L76312 |  | AA | L77243 |  |
| HS35 | D13784 |  | DP | L77237 |  |
| Me15 | L02534 |  | RC | L77244 |  |
| ATK | J02029 |  | RVP | L77239 |  |
| 1503MV | AY818427 |  | PortNn | L77241 |  |
| 2656ND | AY026855 |  | BH223 | AY509602 |  |
| 979MO | AY818423 |  | BH315 | AF032992 |  |
| 1380MV | AY818425 |  | AF032992 | AF032993 |  |
| 1443MV | AY818426 |  |  |  |  |
| 2472LE | AY818430 |  |  |  |  |
| 1842LE | AY818429 |  |  |  |  |
| 2810YI | AY818432 |  |  |  |  |
| 1127MO | AY818433 |  |  |  |  |
| 1259NG | AY818424 |  |  |  |  |
| CA423 | KY928524 |  |  |  |  |
| CA422 | KY928525 |  |  |  |  |
| CA253 | EU108722 |  |  |  |  |
| JPNBr41 | AY499185 |  |  |  |  |

AIC: Akaike information criteria. BIC: Bayesian information criteria.

Table S3. Identification of distinct clusters shown in the phylogenetic trees of HTLV-1 and HTLV-2.

| Virus: Subtype and Subgroup (Figure) | Clusters | Isolate | Identical isolates |
| --- | --- | --- | --- |
| HTLV-1aA (2) | IDUPa539 | IDUPa539 | No identical isolate |
| HTLV-1aA (2) | NIDUPa507+6 | NIDUPa507 | IDUPa603, IDUPa204, NIDUPa369, IDUPa86, NIDUPa124 and NIDUPa46 |
| HTLV-1aA (2) | NIDUPa301+1 | NIDUPa301 | NIDUPa425 |
| HTLV-1aA (2) | IDUPa34+1 | IDUPa34 | IDUPa514 |
| HTLV-1aA (2) | IDUPa257+2 | IDUPa257 | NIDUPa796 and NIDUPa169 |
| HTLV-1aA (2) | IDUPa641 | IDUPa641 | No identical isolate |
| HTLV-1aA (2) | NIDUPa95+2 | NIDUPa95 | NIDUPa168 e NIDUPa98 |
| HTLV-1aA (2) | NIDUPa700+1 | NIDUPa700 | NIDUPa274 |
| HTLV-1aA (2) | NIDUPa19+1 | NIDUPa19 | NIDUPa190 |
| HTLV-1aB (2) | IDUPa609+1 | IDUPa609 | IDUPa618 |
| HTLV-2c (3) | IDUPa210 | IDUPa210 | No identical isolate |
| HTLV-2c (3) | NIDUPa398 | NIDUPa398 | No identical isolate |
| HTLV-2c (3) | IDUPa363 | IDUPa363 | No identical isolate |
| HTLV-2c (3) | IDUPa101 | IDUPa101 | No identical isolate |
| HTLV-2c (3) | IDUPa315+1 | IDUPa315 | IDUPa213 |
| HTLV-2c (3) | IDUPa180+1 | IDUPa180 | IDUPa363 |
| HTLV-2c (3) | IDUPa412 | IDUPa412 | No identical isolate |
| HTLV-2c (3) | NIDUPa224 | NIDUPa224 | No identical isolate |
| HTLV-2c (3) | IDUPa129+1 | IDUPa129 | IDUPa562 |
| HTLV-2c (3) | NIDUPa346 | NIDUPa346 | No identical isolate |
| HTLV-2b (3) | IDUPa404+3 | IDUPa404 | IDUPa90, IDUPa719 and IDUPa281 |
| HTLV-2b (3) | NIDUPa270+1 | NIDUPa270 | NIDUPa157 |

Table S4. Factors or behaviors not associated with HTLV-1/2 in people who use illicit drugs and their subgroups using bivariate analysis.

| Characteristics/behaviors |  | 715 NIDUs | | |  | 111 IDUs | | |  | 826 PWUDs | | |
| --- | --- | --- | --- | --- | --- | --- | --- | --- | --- | --- | --- | --- |
|  |  | *n* NIDUs | *n* PCR+ | OR (95% CI) |  | *n* IDUs | *n* PCR+ | OR (95% CI) |  | *n* PWUDs | *n* PCR+ | OR (95% CI) |
| Male |  | 519 | 14 | 0.9 (0.3 - 2.3) |  | 85 | 15 | 0.4 (0.2 - 1.1) |  | 604 | 29 | 0.7 (0.4 - 1.3) |
| Age over 40 years |  | 70 | 1 | 0.5 (0.1 - 3.6) |  | 40 | 4 | 0.3 (0.1 - 1.0) |  | 110 | 5 | 0.9 (0.3 - 2.2) |
| Heterosexual |  | 650 | 16 | 0.4 (0.1 - 1.2) |  | 103 | 21 | 0.3 (0.1 - 1.7) |  | 753 | 37 | 0.5 (0.2 - 1.1) |
| Single, separated or widowed^†^ |  | 472 | 12 | 0.8 (0.3 - 1.9) |  | 78 | 15 | 0.6 (0.2 - 1.6) |  | 550 | 27 | 0.8 (0.4 - 1.5) |
| Up to 10 years of study |  | 392 | 9 | 0.7 (0.2 - 1.6) |  | 67 | 12 | 0.6 (0.2 - 1.4) |  | 459 | 21 | 0.7 (0.4 - 1.3) |
| Up to one minimum wage per month^†^ |  | 465 | 16 | 2.2 (0.7 - 6.6) |  | 40 | 9 | 1.1 (0.4 - 2.8) |  | 515 | 25 | 0.8 (0.4 - 1.5) |
| Blood transfusion |  | 91 | 3 | 1.2 (0.3 - 4.2) |  | 10 | 3 | 1.6 (0.4 - 6.9) |  | 101 | 6 | 1.1 (0.5 - 2.8) |
| Tattoos |  | 477 | 14 | 1.2 (0.4 - 3.1) |  | 78 | 17 | 1.0 (0.4 - 2.7) |  | 555 | 31 | 1.2 (0.6 - 2.3) |

PWUDs: People who used illicit drugs. NIDUs: people used only non-injecting drugs during their lives. IDUs: People who have used injectable and non-injectable drugs during their lives. ^†^Last 12 months. OR: Odds ratio. CI: Confidence Intervals.
